# Ultrasonograhic Identification of the Entrapment of a Transligamentous Sensory Branch of the Ulnar Nerve

**DOI:** 10.3390/diagnostics16030405

**Published:** 2026-01-27

**Authors:** Wei-Ting Wu, Ke-Vin Chang, Levent Özçakar

**Affiliations:** 1 Department of Physical Medicine and Rehabilitation, Community and Geriatric Research Center, National Taiwan University Hospital, Bei-Hu Branch, Taipei 108206, Taiwan; b40560@bh.ntuh.gov.tw; 2 Department of Physical Medicine and Rehabilitation, National Taiwan University College of Medicine, Taipei 100233, Taiwan; 3 Center for Regional Anesthesia and Pain Medicine, Wang-Fang Hospital, Taipei Medical University, Taipei 110301, Taiwan; 4 Department of Physical and Rehabilitation Medicine, Hacettepe University Medical School, Ankara 06100, Turkey; lozcakar@hacettepe.edu.tr

**Keywords:** wrist, ligament, paresthesia, aberrant nerve, ultrasound

## Abstract

Anatomical variations of the ulnar nerve at the wrist are uncommon and may lead to diagnostic confusion or iatrogenic injury if unrecognized. We present an ultrasound-based identification of a rare transligamentous ulnar nerve sensory branch entrapment in an elderly male with chronic ulnar-sided hand paresthesia. High-resolution ultrasonography revealed an aberrant sensory branch deviating from the ulnar nerve, piercing the palmar carpal ligament, and coursing superficially rather than entering Guyon’s canal. Further assessment demonstrated focal nerve flattening within the ligament with proximal enlargement, consistent with entrapment. This case highlights the value of ultrasound in detecting rare peripheral nerve variants and their entrapments. Therefore, it is also noteworthy to extend the sonographic evaluation beyond conventional entrapment sites at the wrist.

**Figure 1:**
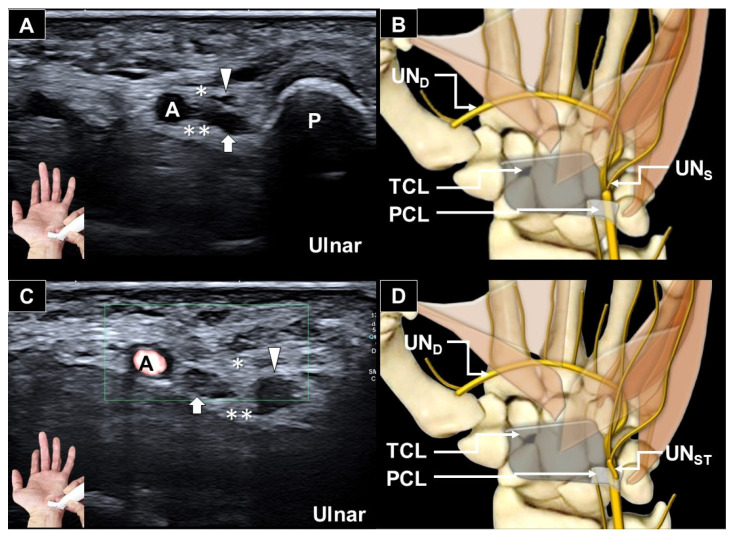
Ultrasonography of the ulnar nerve within Guyon’s canal in the short-axis view demonstrates a normal appearance on the asymptomatic side ((**A**), ultrasound image; (**B**), schematic illustration). On the symptomatic side, the sensory branch of the ulnar nerve shows ulnar deviation within Guyon’s canal ((**C**), ultrasound image; (**D**), schematic illustration). White arrow and UN_D_: deep motor branch of the ulnar nerve; white arrowhead and UN_S_: superficial sensory branch of the ulnar nerve; UN_ST_: transligamentous superficial sensory branch of the ulnar nerve; A: ulnar artery; P: pisiform; double asterisks and TCL: transverse carpal ligament; asterisk and PCL: palmar carpal ligament.

**Figure 2:**
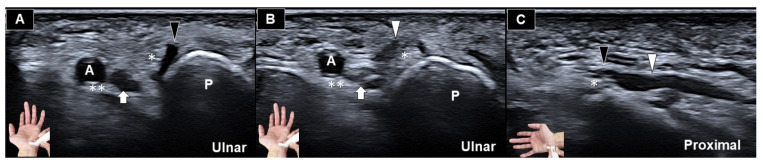
Ultrasonography (short-axis view) demonstrated a transligamentous course of the ulnar nerve sensory branch which pierced the palmar carpal ligament (**A**) and coursed superficially/radially over the canal (**B**). The long-axis view demonstrated flattening of the nerve fascicle due to intraligamentous compression (**C**). White arrow: deep motor branch of the ulnar nerve; black arrowhead: superficial sensory branch of the ulnar nerve coursing within the ligament; white arrowhead: superficial sensory branch of the ulnar nerve distal to the site of compression; A: ulnar artery; P: pisiform; double asterisks and asterisk. The two ultrasonographic images (Figure 1 and Figure 2) were obtained from a 72-year-old male farmer (height: 166 cm; weight: 73 kg) who presented with chronic paresthesia and intermittent pain along the ulnar aspect of the hand, predominantly involving the ring and small fingers for one year. His occupation required prolonged gripping of farming tools and repetitive branch cutting, during which the symptoms progressively worsened. The visual analog scale score for pain was 5 on a 10-cm scale. Prior treatments—including oral nonsteroidal anti-inflammatory drugs and physical therapy (transcutaneous electrical nerve stimulation and laser)—had been ineffective. Symptoms were exacerbated by wrist flexion and nocturnal compression. Physical examination revealed a positive Tinel sign over the ulnar side of the wrist. Electrodiagnostic testing demonstrated distal ulnar sensory neuropathy without evidence of cubital tunnel involvement. As the electrophysiological findings for Guyon’s canal syndrome were inconclusive, ultrasound examination was undertaken to investigate a distal source of ulnar nerve irritation. Ultrasound examinations were performed using a 5–17 MHz linear transducer (Aplio i700 Canon Medical Systems Corp., Otawara, Japan). Initially, the transducer was positioned over the palmar carpal ligament in the short-axis plane, i.e., to evaluate the morphology of the superficial sensory and deep motor branches of the ulnar nerve. Compared with the contralateral side, the symptomatic wrist demonstrated an aberrant sensory branch arising from the ulnar nerve. It pierced the distal portion of the palmar carpal ligament and coursed superficially over the ligament—rather than entering Guyon’s canal. The affected nerve appeared flattened within the ligament, accompanied by focal enlargement distal to the compression site. As sono-palpation reproduced the patient’s symptoms, the scenario was considered to be transligamentous entrapment of the sensory branch of the ulnar nerve. In contrast, the deep branch of the ulnar nerve appeared to be normal. In contrast to the more commonly described anatomical variations of the recurrent motor branch of the median nerve [1,2,3,4,5], the transligamentous course of the ulnar nerve sensory branch is exceedingly rare and has been sparsely reported in the literature (as intraoperative observations) [6,7]. Guyon’s canal syndrome is relatively uncommon in clinical practice, and this specific anatomical variant appears to be exceedingly rare even among symptomatic patients. To date, the literature has primarily described anatomical variations of the ulnar nerve related to branching patterns (e.g., bifurcation or trifurcation), interconnections with the median nerve (e.g., Berrettini anastomosis), and variants of re-connection between the dorsal ulnar cutaneous nerve with the main trunk of the ulnar nerve (e.g., Kaplan anastomosis). However, only a single case report has documented a sensory branch of the ulnar nerve traversing the palmar carpal ligament [6]. In that report, the patient presented with numbness involving all digits of the right hand, most prominently affecting the ring and small fingers, along with frequent nocturnal pain. Consequently, no prevalence data or epidemiological statistics are currently available in the literature. The present report is the first to identify this rare anatomical variant using ultrasonography, providing a novel diagnostic perspective and broadening the clinical framework for differentiating ulnar nerve compression at the wrist. In the future, cadaveric studies could be conducted to estimate the prevalence of this variant. Ultrasonography is uniquely suited to detect such variants [8]: it enables real-time visualization of small nerve fascicles [9,10,11] and their relationships with surrounding ligaments (e.g., pisohamate, pisometacarpal, transverse carpal, and palmar carpal ligaments) [12]. In addition, space-occupying lesions such as scars, ganglia [13], lipomas, anomalous heads of the abductor digiti minimi muscle [14,15,16], or tophi can be readily identified. Not to mention, dynamic changes during wrist motion can also be assessed [17]. This case highlights a subtle and frequently overlooked cause of Guyon’s canal syndrome [12,18,19], emphasizing the importance of accurate recognition to avoid misdiagnosis. Awareness of this variant is also essential during invasive procedures (e.g., carpal tunnel release, palmar carpal ligament release for Guyon’s canal syndrome, ulnar nerve hydrodissection at this level using either in-plane or out-of-plane approaches, or flexor carpi ulnaris tendon injection) [20] to minimize the risk of iatrogenic injury. For cases involving this anatomical variant, minimally invasive microsurgical release of the palmar carpal ligament represents a more appropriate therapeutic option. If injection therapy is planned, a radial-to-ulnar needle approach may be considered to allow intraligamentous hydrodissection while avoiding injury to the ulnar artery. Lastly, extending the ultrasound examination beyond the conventional entrapment sites would also be noteworthy for identifying rare but clinically significant nerve variants at the wrist.

## Data Availability

The original contributions presented in the study are included in the article; further inquiries can be directed to the corresponding author.
